# Feasibility of Sexual Health and Contraceptive Web Services for Adolescents and Young Adults: Retrospective Study of a Pilot Program on Reunion Island

**DOI:** 10.2196/52557

**Published:** 2024-11-01

**Authors:** Danielle Reynaud, Nicolas Bouscaren, Emmanuelle Cartron, Catherine Marimoutou

**Affiliations:** 1 Department of Nursing, Rehabilitation and Medical Techniques (South Site), University Hospital Center, BP 350 avenue François Mitterand, Terre Sainte, Saint-Pierre, Reunion Island, 97410, Reunion, 262 0262 35 90 00 ext 55214; 2 Centre d'Investigation Clinique 1410 - INSERM, University Hospital Center, Saint-Pierre, Reunion; 3 Unité de soutien méthodologique de gestion et d’analyse de données, DRCI, University Hospital Center Reunion Island, Saint-Pierre, Reunion; 4 Université Paris Cité, INSERM, ECEVE, Paris, France; 5 Health Department, University Hospital Center, Saint-Denis, Reunion Island

**Keywords:** sexual health, adolescent, young adults, web application, contraception prescription, contraception, teleconsultation, telemedicine, youth, usage, e-consultation, web based

## Abstract

**Background:**

Sexual health indicators for adolescents and young adults (AYAs) aged between 13 and 25 years are particularly poor on Reunion Island. Access to accurate information as well as sexual health and contraceptive services are vital to maintaining sexual well-being. Teleconsultations offer a promising approach to addressing the sexual health and contraceptive needs of AYAs who are more susceptible to engaging in unprotected sexual intercourse. However, the literature on digital sexual health services for this demographic group is limited.

**Objective:**

This study aims to describe the feasibility of a pilot sexual health and contraceptive teleconsultation web service used by AYAs on Reunion Island.

**Methods:**

A descriptive, retrospective study was conducted at the Reunion Island University Hospital Center using a convenient sample. Eligible participants were informed about the program through various communication channels, including seminars for health care professionals, radio broadcasts, posters, flyers, press articles, videos, and social media posts. AYAs accessed a web-based platform named SEXTUOZE from December 15, 2021, to September 30, 2022, that offered sexual health information and teleconsultations. Data collected included participant and teleconsultation characteristics, patient satisfaction, and the quality of completeness of medical records.

**Results:**

A total of 22 teleconsultations were scheduled and 7 were completed, all via synchronous video communication (duration: median 35 min). Overall, 4731 sessions were generated on the SEXTUOZE website. Reasons cited for accessing the web services were to seek sexual health advice (8/22, 36%), receive an initial birth control prescription (12/22, 55%), and inquire about condom prescriptions (2/22, 9%).

**Conclusions:**

While teleconsultation use for sexual health was initially low, it rose toward the end of the study period. Considering all elements of the implementation theory, future research should design interventions that not only are more operative and tailored but also ensure their adoption and sustainability in various health contexts.

## Introduction

Adolescent pregnancy, defined as pregnancy in adolescents and young adults (AYAs) younger than 20 years, is a global public health concern occurring in high-, middle-, and low-income countries [1]. According to the World Health Organization, complications in pregnancy are the leading cause of death for girls aged 15 to 19 years [2]. These pregnancies are influenced by various interconnected factors, including sociocultural norms, lack of sexual education, poor economic conditions, and relationship dynamics, as well as limited access to reproductive health services. Lack of access to and use of contraceptive methods has been considered a key factor [1]. Social and family pressures, as well as media influences, also play a significant role [3].

On Reunion Island, a French overseas department situated in the Indian Ocean, unwanted pregnancies are a significant concern [4]. In 2022, according to the French National Institute of Statistics and Economic Studies (INSEE), 4.3% of births involved mothers younger than 20 years, a rate almost 3 times higher than that of mainland France (1.7%) [5]. The abortion rate was 13%, double that of mainland France [6]. Abortion rates were also high among young people aged 20 to 24 years, with 26.9% in metropolitan France and the overseas territories [6]. Moreover, data from the regional health observatory in 2019 reported that only half of sexually active AYAs aged 15 to 17 years used contraception during their first sexual encounter [7].

For young women aged 15 to 25 years who had already had their first sexual intercourse, a survey reported that 56% used a contraceptive method other than condoms and that a lack of knowledge and negative perceptions of contraception, along with the family taboo surrounding sexuality, contributed to it [7]. Some AYAs were aware of various contraceptive methods; however, their fragmented understanding of how to obtain them prevented the effective prevention of unwanted pregnancies [8]. de Pirey et al [9] presented that nearly a quarter of Reunionese minors who had an abortion or gave birth in 2009 never used any contraceptive method and that 42% did not use any contraceptive method in the month their pregnancy began. This study also reported that these minors had difficulties in accessing contraception, citing financial or confidentiality issues [9].

Sexual health resources available to AYAs on Reunion Island are substantial; however, they may be hindered by the mountainous geography and the isolated nature of many villages [10]. Patient access to sexual health services, particularly for AYAs who have scheduling constraints such as attending school or who do not drive, may be significantly impacted. This phenomenon may also be aggravated by the heterogeneous distribution of health care services on the island [10]. There are 3 free drop-in sexual health centers (known as the Centres Gratuits d’Information, de Dépistage et de Diagnostic [CeGIDD]) and 10 family planning and education facilities (Centres de Planification et d’Éducation Familiale [CPEF]) located on the island, which offer free birth control consultations and sexually transmitted illness (STI) screenings for AYAs. Contraception is free of charge and can be provided without informing the parents of minors [10]. Moreover, digital coverage extends to 80% of the territory, and in 2017, a total of 78% of the population older than 15 years were reported to have internet access at home [11].

Telemedicine activities, such as teleconsultations, are a promising approach to improving access to health care services [12]. Telemedicine is the use of telecommunications technology to provide medical care, consultations, and health education remotely. It can include services such as web-based doctor visits, remote monitoring of patients, digital transmission of medical data, and the provision of health information via electronic communication. The aim of telemedicine is to enhance the accessibility and quality of health care, particularly for individuals in remote or underserved areas, by overcoming the physical distance between patients and health care providers [12].

In gynecologic care, telemedicine may provide an innovative solution for improving access to sexual health services [13-15]. Globally, teleconsultations allow access to contraceptive care [16], birth control advice [17], medication-induced abortions [18], and the management of STIs [19]. However, access to sexual health care and contraceptive methods faces several contemporary challenges, particularly concerning teleconsultation services for AYAs. Literature has reported the frequency of internet connection failures, lower acceptability of such services, confidentiality, and data security concerns among a younger audience while confronted by the lack of data on the number and satisfaction of teleconsultations carried out [20]. Patients consulting gynecologists via digital health services in Australia reported satisfaction with their experience, finding it useful, time-saving, and cost-efficient [21]. In another study, although face-to-face care was preferred, digital health services for AYA contraceptive needs were perceived as both acceptable and accessible by most patients [19]. A study on young adults examined how AYAs used contraceptive methods over time, based on telemedicine consultations with health care professionals [22]. The implementation of teleconsultations for sexual and reproductive health in isolated regions, such as Reunion Island, presents a unique opportunity to overcome geographical and sociocultural barriers. Few studies have explored the impact of such solutions in insular contexts, providing valuable insights into the adaptation and implementation of these services in similar regions around the world. The objective of this study was to evaluate the feasibility of a sexual health teleconsultation website focused on providing detailed information and issuing contraceptive prescriptions for AYAs on Reunion Island.

## Methods

### Study Design

A retrospective single-center analysis of a sexual health pilot program implemented at the University Hospital of Reunion Island was conducted from December 15, 2021, to September 30, 2022. In June 2020, the pilot program consisted of two phases: (1) designing a website and teleconsultation platform (SEXTUOZE) within a Symphony Framework environment, and (2) evaluating the feasibility of the teleconsultations. A 24-month budget was allocated to complete the project; 14 months were required for the website development due to administrative, technological, and institutional issues, leaving only 10 months for testing with AYAs. The platform was developed by a team comprised of midwives, nurses, doctors, and the primary investigator, in collaboration with the development team. Due to time constraints, AYAs were not invited to participate in the setup process. Multimedia Appendix 1 details how SEXTUOZE worked. The implementation of the teleconsultation platform responded to a governmental request as part of the sexual health strategy in France [23]. The study followed the Strengthening the Reporting of Observational Studies in Epidemiology (STROBE) guidelines.

### Publicity

Information about the pilot study and SEXTUOZE was disseminated through seminars for 150 health care professionals, radio broadcasts, posters, flyers, news articles, videos, and social media posts. Two hundred students attended the presentations at their schools. Content was shared on 2 social media networks (Facebook and Instagram) under the hashtag #Sextuoze. Participating nurses and midwives managed the sites after specific training. Weekly publications and daily stories were posted throughout the 10-month study period, following specific hospital guidelines. The various media campaigns provided information about the digital health services, their mission, and how they worked to encourage AYAs to use sexual health and contraceptive web-platform services.

### Sampling and Recruitment

We used convenience sampling, including all AYAs aged 15 to 25 years residing on Reunion Island, who accessed the SEXTUOZE teleconsultation platform during the study period. Potential participants were informed about the program through various communication channels, including seminars for health care professionals, radio broadcasts, posters, flyers, press articles, videos, and social media posts (Facebook and Instagram).

### Study Intervention

The teleconsultations concerned information and education about sexuality (including prevention of STIs, the morning-after pill, birth control, and prevention of sexist behavior and sexual violence) as well as birth control (including first-time prescriptions for young females and condoms). The 3-month follow-up after birth control initiation (as per French Health Authority guidelines) was also offered. AYAs had the opportunity to participate in teleconsultations with midwives using a computer or smartphone. Teleconsultations were available 6 days a week from Monday to Friday (6 AM to 7 PM) and on Saturday from 6 AM to noon. A total of 1000 consultation slots were offered during the study period. Once an account was created, AYAs had direct access to the appointment scheduling system and could register for the time slot that suited them. Appointments up to 2 hours before a teleconsultation were possible. A teleconsultation was free of charge and adhered to privacy and confidentiality laws. Patients received a link via email both 1 day prior and again a few minutes before their appointment. Teleconsultations could be carried out with the patient alone or in the company of a primary caregiver. At the end of each teleconsultation, participants were invited to answer a Likert-scale satisfaction question: “On a scale of 1 to 5, how would you rate your overall satisfaction with this teleconsultation? (1: very dissatisfied; 2: dissatisfied; 3: neutral; 4: satisfied; 5: very satisfied).” After 24 hours, participants received an online satisfaction evaluation form as well.

### Study Variables

The study variables examined included scheduled teleconsultation appointments, the total number of completed teleconsultations, the mean duration of teleconsultations, website traffic, patient satisfaction, and the quality and completeness of the medical files.

### Data Collection

An Excel (Microsoft Corporation) file was created for data to store account creation and appointment scheduling information. Patient characteristics, including age, sex, socio-professional data, contraceptive prescription, medical history, and satisfaction, were extracted from their electronic medical records.

### Statistical Analysis

Descriptive statistics were expressed in numbers and percentages, including the median. Statistical analyses were performed using SAS (version 9.4; SAS Institute Inc).

### Ethical Considerations

In accordance with French regulations, we received informed consent from each patient (by telephone) before accessing their medical files. This process ensured that participants understood the study’s objectives, the nature of the data collected, and their rights to confidentiality. This study was approved by the Research Ethics Committee of the University Hospital of Bordeaux, France (reference number: CER-BDX 2023‐74) and was registered with the National Institute of Health Database (#F20220518181737).

## Results

### Website Traffic

**Figure 1.**
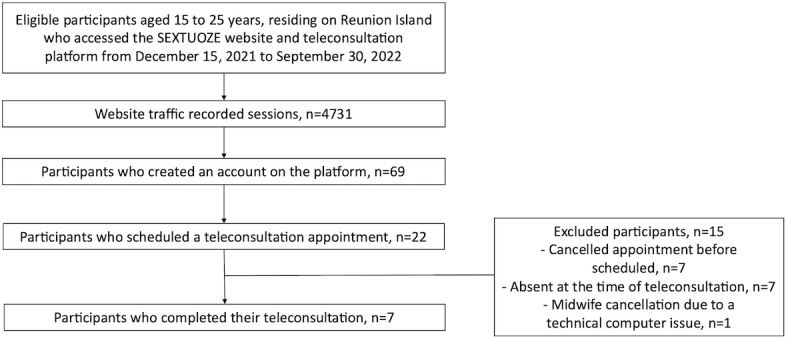
Study flowchart.

**Figure 2.**
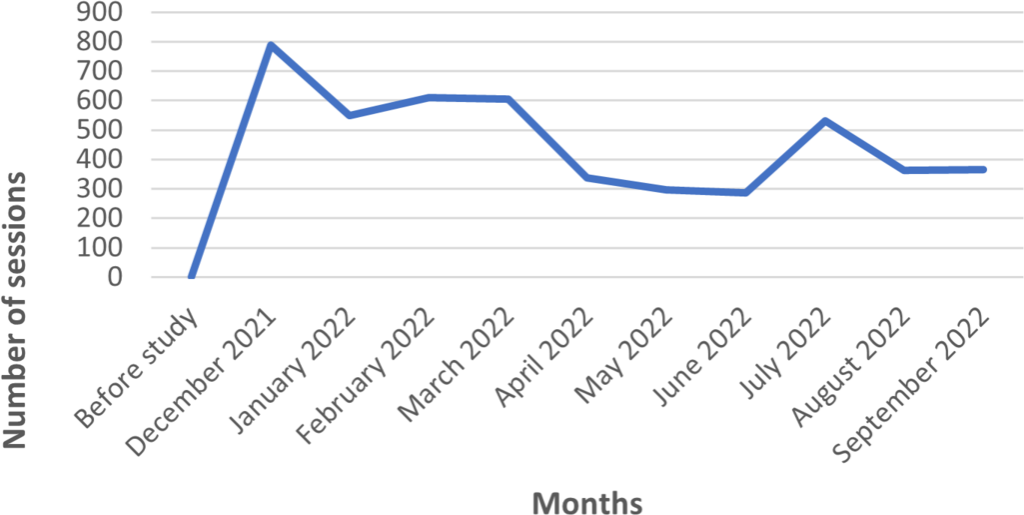
Monthly website traffic data from December 2021 to September 2022 on Reunion Island.

**Figure 3.**
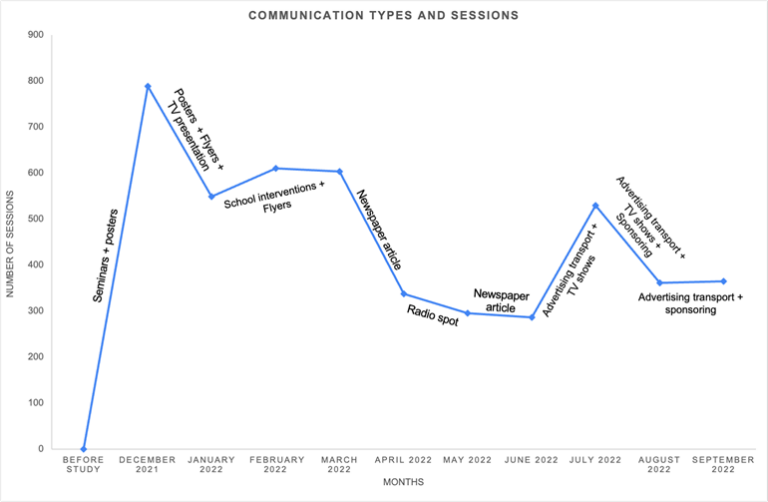
Impact of communication campaign on website traffic. The campaign was to promote the use of a teleconsultation platform for sexual health services to adolescents and young adults residing on Reunion Island.

Overall, 4731 sessions were recorded on the platform, including visits, whether to consult information on sexual health and contraception or to explore the teleconsultation services offered (Figure 1). Out of these sessions, 69 unique users created an account on the platform to access the teleconsultation services, among which 22 scheduled a teleconsultation appointment. The mean number of monthly sessions was 473 sessions per month, with the highest at 789 sessions at the start of the study and the lowest at 287 sessions in June 2022 (Figure 2). These variations may have been related to both the general and targeted messaging of communication campaigns. Presentations in schools, combined with the distribution of posters and flyers, were the most effective means of promotion, as indicated by the increase in website traffic. Radio broadcasts did not seem to positively influence website traffic (Figure 3).

### Sexual Health and Contraceptive Teleconsultations

A total of 22 AYAs scheduled a teleconsultation appointment. Of these, 7 (32%) AYAs completed their consultation, 7 (32%) AYAs canceled before the scheduled date, 7 (32%) AYAs were absent at the time of teleconsultation, and in one case, a midwife canceled due to a technical computer issue. Among the scheduled consultations, 55% (n=12) were for an initial birth control prescription, while 36% (n=8) were for sexual health advice. Consultations related to inquiring about condom prescriptions represented 9% (n=2) of cases. No birth control follow-up consultation was scheduled. Completed teleconsultations were conducted via videoconferencing and lasted for a median duration of 35 minutes. Among the 7 patients who completed teleconsultation, the median age was 18 years, and all were females who resided in the northern and southern regions of Reunion Island. Various birth control methods were prescribed, including pills, implants, patches, and intrauterine devices (Table 1).

**Table 1.** Patient and teleconsultation use characteristics.

Variables		Value
Age (years; N=7), median (IQR)		18.0 (15.0-24.0)
Sex (female), n (%)		7 (100)
**Education level**		
	Secondary (high school)	—^a^
	Tertiary (university)	—
**Region of residence**		
	Northern region of Reunion Island	—
	Southern region of Reunion Island	—
Living with primary caregiver/parent		—
Teleconsultation duration, median (IQR)		35.0 (25.0-60.0)
**Reason for teleconsultation**		
	Sexual health counseling	—
	First contraception prescription	—
Contraception prescribed		—
**Type of prescribed contraception**		
	Intrauterine device	—
	Implant	—
	Pill	—
**Experience of teleconsultation, n (%)**		
	Very satisfactory	5 (71)
	Satisfactory	2 (29)

a —: Confidential.

### Patient Satisfaction

Participants revealed high levels of self-perceived satisfaction with the use of teleconsultation for contraceptive care. Most AYAs expressed positive feedback, with 5 out of 7 reporting that they were very satisfied and 2 out of 7 indicating they were satisfied with their teleconsultation experience. It should be noted that the satisfaction forms sent online were not returned by any of the teleconsultants.

### Quality and Completeness of Medical Files

The survey on the use of teleconsultation for contraceptive care among AYAs demonstrated complete compliance with the required information submission. All necessary data for the teleconsultations were successfully provided by the participants, ensuring that the process was thorough and efficient.

## Discussion

### Principal Findings

To our knowledge, this is the first study to assess the feasibility of a sexual health and contraceptive teleconsultation platform on Reunion Island. Establishing this platform necessitated the collaboration of a multidisciplinary team to navigate administrative, technological, and institutional challenges. While the number of completed teleconsultations was low, website traffic results indicated a notable interest in digital health care. The low use of teleconsultations for obtaining contraception was also observed in an American study by Yarger et al [22], wherein only 6% of 1630 young adults declared teleconsultation use. A lack of collaboration between researchers and stakeholders, particularly AYAs, to gather information on their needs and determine the optimal approach to implement and communicate interventions may explain the low use of teleconsultations observed in our study. Active participation of stakeholders, including children, parents, and health care providers, at all stages of developing a health intervention is essential to increase the effective use of health care interventions [24,25]. Co-design, as defined by Sanders and Stappers [26], involves collective creativity throughout the design process and can potentially lead to the development of more engaging, satisfying, and useful interventions for potential end users. Moreover, cocreation, practiced at the early front end of the design development process, can have positive, long-term effects. Involving AYAs in the co-design of the platform, according to cocreation principles, may have improved outcomes. Despite efforts to promote the program and sexual health and contraceptive websites to both health professionals and the general public, very few AYAs were informed about the program, which was crucial to reaching them.

Social media networks such as Instagram and Facebook social media networks were used to inform the target group about SEXTUOZE during the study period because these platforms are widespread among AYAs and are considered to be important tools in promoting sexual health [27,28]. On the other hand, when combined with other distribution channels, they did not result in providing significant awareness among AYAs. This finding aligns with existing literature, such as a study by Lim et al [29], which indicated that many young adults in Australia were not comfortable accessing health information through social media channels. In hindsight, our communication strategy may not have been sufficiently captivating or motivating to achieve the expected results. Employing a community manager may have been more effective in developing a successful strategy.

Considering that sexual behavior is a sensitive matter that can lead to social stigma, it is well established that confidentiality is a key element in optimally functioning sexual and reproductive health care services [30]. Confidentiality protection encourages AYAs to autonomously seek care and share sensitive information necessary for providing appropriate care [31]. Studies have shown that many AYAs are unaware of the specific privacy safeguards concerning the transmission of sexual health care information in place [32,33]. However, the understanding of confidentiality may vary according to age, with young adolescents being less aware of their right to confidentiality [33]. It is essential to make it clear to young adults that their privacy will be ensured, even when it is through digital communication. This is especially relevant given that most countries are moving toward integrated health care models with electronically accessed information that connects existing health care provider structures, regional clinical result databases, and the sharing of patient records [34]. For example, a study conducted in the United States found that 88% of adolescents were more inclined to seek sexual health services if they were assured of their privacy and confidentiality [35]. This underscores the significance of transparent communication regarding privacy policies and the implementation of robust data protection measures. Such measures are crucial for fostering trust and encouraging AYAs to use these services without fear of exposure or judgment.

Analyzing the causes behind why participants dropped off at different stages (such as canceling the scheduled appointment and not showing up) allowed us to identify specific friction points. For example, technical issues and a lack of familiarity with teleconsultations were 2 such reasons in this study. On Reunion Island, among those aged 15 to 44 years, 9 out of 10 have high-speed internet at their residence. However, those residing in the highlands of the island are less well equipped, with only 70% having internet access at home and 58% having high-speed internet [11]. By addressing these obstacles, we can propose targeted solutions to improve service adoption in the future. This analytical approach, which focused on optimizing user processes, can be applied to other contexts and teleconsultation use services, providing valuable insights to enhance the efficiency and acceptability of digital health solutions.

### Limitations

Our study had several limitations. First, the small sample size limited the generalizability of our findings. The low number of completed teleconsultations may not have represented the broader population of AYAs on Reunion Island. Second, self-selection bias may have influenced the results, since those who chose to participate might have differed from those who did not in terms of motivation and access to digital resources. Third, the short duration of the evaluation period may not have been sufficient to capture the full potential and challenges of implementing the teleconsultation service. Changes in behavior take time and are a complex and iterative process that individuals who are aware of best health practices do not always succeed in adjusting [36]. Last, the lack of returned satisfaction forms restricted our understanding of patient experiences and satisfaction. Future research should aim to include larger and more diverse samples to enhance the generalizability of findings. Longitudinal studies would be beneficial to assess the long-term impact and sustainability of teleconsultation-based sexual health services. Additionally, strategies to improve awareness and engagement, such as involving community managers and leveraging popular social media platforms such as TikTok, should be explored. Ensuring digital literacy and addressing barriers to internet access are crucial for equitable implementation. For example, implementing community-based digital literacy programs could help bridge the digital divide. Moreover, maintaining robust confidentiality and privacy measures will be essential to building trust and encouraging the use of teleconsultation services among AYAs. Policy makers should consider supporting teleconsultation initiatives through funding and regulatory frameworks to ensure their sustainability and effectiveness.

Our study also may have highlighted several key lessons that can be used for future initiatives. First, stakeholder engagement is critical for the success of teleconsultation services. Involving AYAs in the design process could have enhanced the usability and acceptance of the platform. Second, effective time management is crucial to ensure timely development and evaluation. Our pilot program had a budget allocated for 24 months to develop and evaluate the platform. However, due to unforeseen administrative and public procurement obstacles, the development phase took longer than initially planned (14 mo) instead of the projected 6 months. Consequently, we were left with only 10 months to conduct the evaluation. Therefore, delays in development can significantly reduce the time available for evaluation and optimization. Third, a well-thought-out awareness and engagement campaign is essential for encouraging the target population to use the services. Robust data confidentiality and protection measures are also necessary to build trust and encourage use among AYAs.

### Conclusion

Our study demonstrates the feasibility and challenges of implementing a teleconsultation platform for sexual health in an isolated region. SEXTUOZE was functional and authorized prescriptions for contraception and sexual health advice and information; however, the demand for teleconsultations was low, and no follow-up or request for emergency contraception was made. By identifying friction points and proposing improvements, we provide valuable insights that can be applied to other similar contexts. This innovative approach, both conceptually and empirically, can contribute to the international body of literature and may underscore the importance of future research to develop more effective, tailored, and sustainable interventions.

## Supplementary material

10.2196/52557 Multimedia Appendix 1 Functions of the sexual health website and teleconsultation platform (SEXTUOZE) used on Reunion Island.

## Abbreviations

AYA: adolescent and young adult
CeGIDD: Centres Gratuits d’Information, de Dépistage et de Diagnostic
CPEF: Centres de Planification et d’Éducation Familiale
INSEE: Institute of Statistics and Economic Studies
STI: sexually transmitted illness
STROBE: Strengthening the Reporting of Observational Studies in Epidemiology
