# Supplementary figures and images for: Feasibility of Sexual Health and Contraceptive Web Services for Adolescents and Young Adults: Retrospective Study of a Pilot Program on Reunion Island
Source: JMIR Pediatr Parent. 2024 Nov 1;7:e52557. doi: 10.2196/52557 (PMC11548867; doi:10.2196/52557)

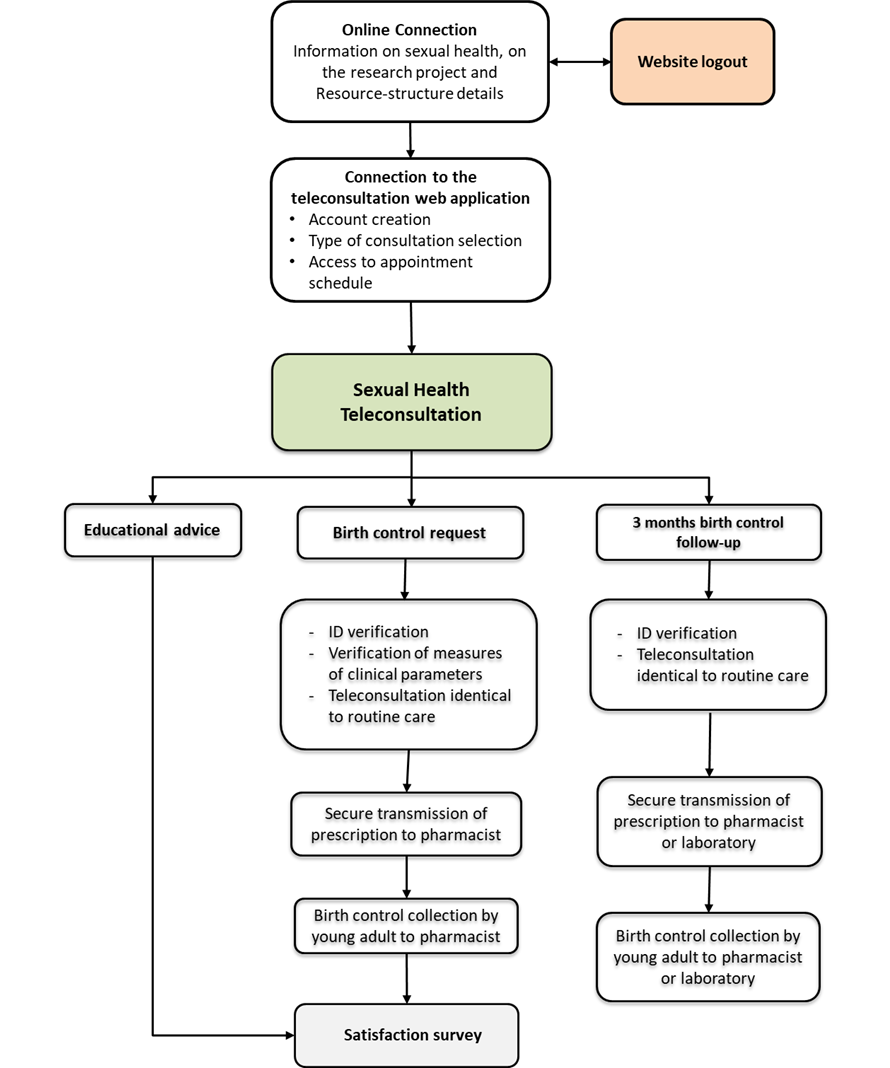

Supplement: Multimedia Appendix 1 [file pediatrics-v7-e52557-s001.png]
